# Supplementary material for: 4-Pyridone-3-carboxamide-1-β-D-ribonucleoside Reduces Cyclophosphamide Effects and Induces Endothelial Inflammation in Murine Breast Cancer Model
Source: Int J Mol Sci. 2024 Dec 24;26(1):35. doi: 10.3390/ijms26010035 (PMC11719935; doi:10.3390/ijms26010035)
Supplement: Supplementary file 1 [file ijms-26-00035-s001.zip › ijms-3335925-supplementary.pdf]

SUPPLEMENTARY MATERIALS

Materials and methods

4-Pyridone-3-carboxamide-1-β-D-ribonucleoside (4PYR). For the ribosylation the Vorbrüggen protocol was used. 5 ml of hexamethyldisilazane (HMDS), and trimethylchlorosilane (TCS) (0.76 ml, 6 mmol) under argon atmosphere, were added to 4PY (276 mg, 2 mmol). Then, the mixture was heated (120°C, 2 h).The resultant mixture was left to cool to room temperature and was co-evaporated with toluene under reduced pressure (3 x 10 ml). In the next step, dry 1,2-dichloroethane (6 ml) was added to the persilylated amide, then the solution of 1,2,3,5-tetra-O-acetyl-β-D-ribofuranose (636 mg, 2 mmol) in 10 ml of 1,2-dichloroethane was added and after that solution of trimethylsilyl triflate (TMSTfl) (0.42 ml, 2.3 mmol) in 5 m of 1,2-dichloroethane. The resulting solution, following 5 h of stirring at 45–50°C, was cooled to 0°C and placed into an ice-water-mixture of saturated aq $NaHCO_3$ solution (25 ml) and CH_2Cl_2 (20 ml), filterd using a pad of Celite and layers separated. The aqueous layer was then extracted with CH2Cl2 (4 x 10 ml). Combination of organic extracts were washed with saturated NaCl solution and dried over Na_2SO_4. After solvents evaporation, the precipitate was purified by column chromatography (silica gel, chloroform/methanol 100:1→4) to obtain an oil, which was crystallized from CH_2Cl_2/pentane affording 1-(2′,3′,5′-tri-O-acetyl-β-D-ribofuranosyl)-4-pyridone-3-carboxamide (565 mg, 71%), white powder, m.p. 199-201°C, lit. 198-201°C.

SUPPLEMENTAL FIGURES

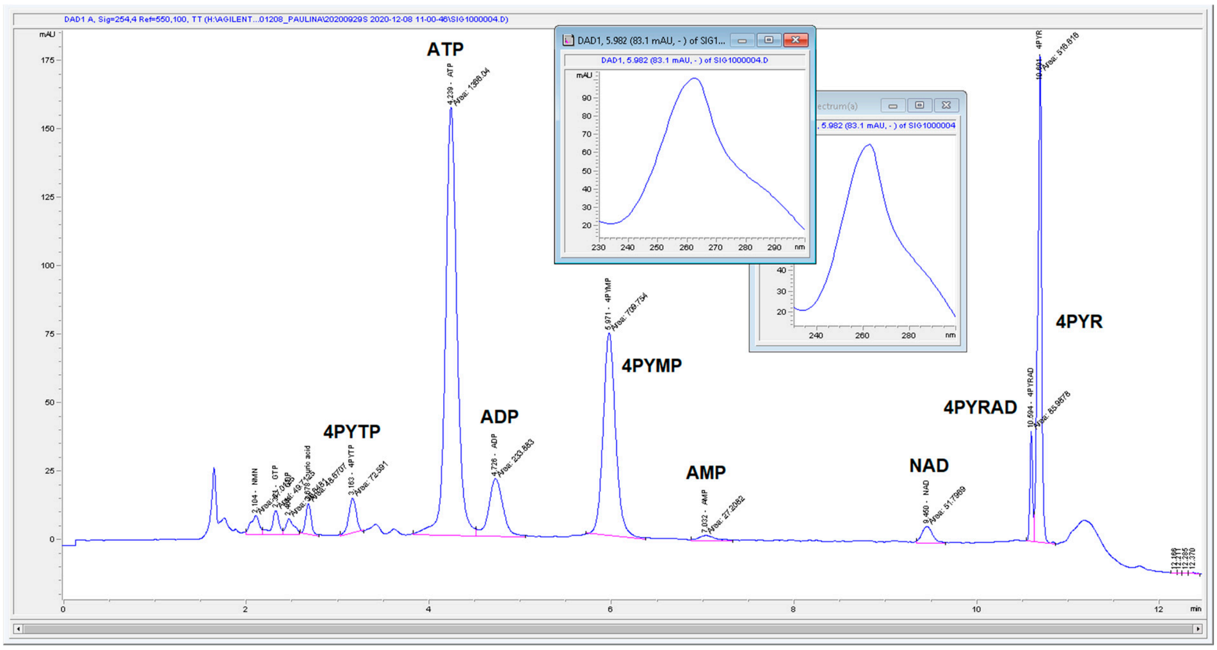

Figure S1. Example from the chromatographic analysis of 4-pyridone-3-carboxamide-1-β-D-ribonucleoside (4PYR) in blood sample and its derivatives with the characteristic for them UV spectrum: 4-pyridone-3-carboxamide-1-β-D-ribonucleoside triphosphate (4PYTP); 4-pyridone-3-carboxamide-1-β-D-ribonucleoside monophosphate (4PYMP) and NAD+ analog - 4PYRAD.

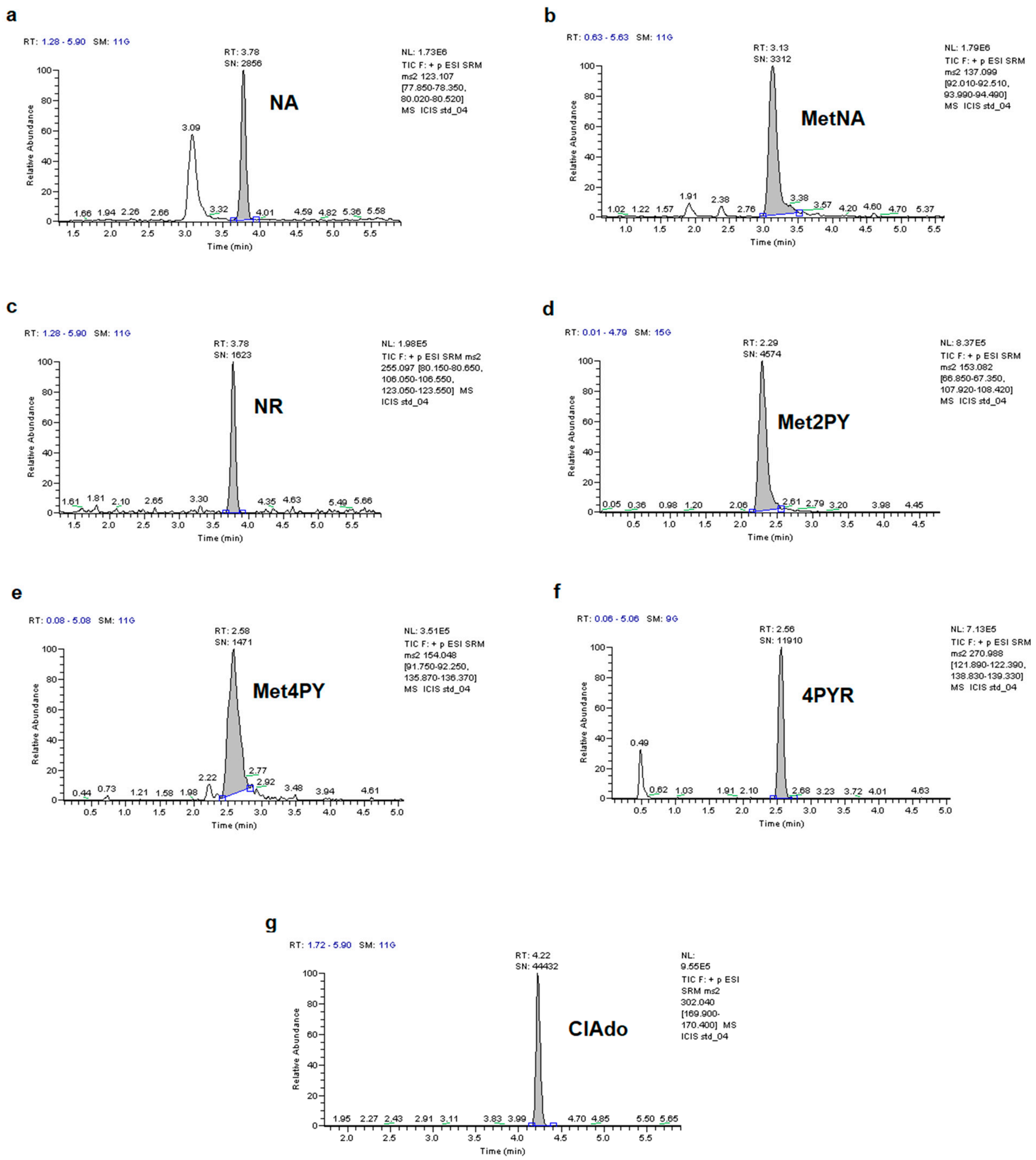

Figure S2. Examples of the LC/MS analysis of nicotinamide metabolites in serum. a) nicotinamide (NA); b) N-methylnicotinamide (MetNA); c) nicotinamide riboside (NR); d) N-methyl-2-pyridone-5-carboxamide (Met2PY); e) N-methyl-4-pyridone-3-carboxamide (Met4PY); f) 4PYR and g) 2-Chloroadenosine (ClAdo) which is used as the internal standard. Optimal collision energies and fragmentation ions were established during continuous infusion of standard solutions into mass spectrometer. Differentiation of two isomers Met2PY and Met4PY was based on specific retention times and fragmentation patterns. For Met2PY observed fragment at m/z=108.2 most likely resulted from loss of carboxamide group. For Met4PY observed fragment at m/z=136.2 most likely resulted from loss of water.
